# Parkinson's disease and *Porphyromonas gingivalis* levels: a case-control study

**DOI:** 10.1590/1678-7765-2025-0780

**Published:** 2026-03-23

**Authors:** Nathália Alves Gusmão, Augusto Paixão Moraes Mateus, Victor Bento Oliveira, Luís Otávio de Miranda Cota, Rafael Paschoal Esteves Lima, José Roberto Cortelli, Sheila Cavalca Cortelli, Fernando Oliveira Costa

**Affiliations:** 1 Universidade Federal de Minas Gerais Faculdade de Odontologia Belo Horizonte Brasil Universidade Federal de Minas Gerais, Faculdade de Odontologia, Belo Horizonte, Brasil.; 2 Universidade de Taubaté Taubaté Brasil Universidade de Taubaté, Taubaté, Brasil.

**Keywords:** Case-control study, Neurodegeneration, Parkinson’s disease, Periodontitis, Systemic inflammation, Porphyromonas gingivalis

## Abstract

Neurodegenerative diseases have been associated with periodontal diseases.

**Objectives:**

This study aimed to evaluate the clinical and epidemiological aspects of the association between Parkinson's disease (PKD) and periodontitis (PE), including the quantification of *Porphyromonas gingivalis* and the influence of different risk variables on this association.

**Methodology:**

This case-control study included 172 individuals: 86 cases (PKD) and 86 controls (without any neurodegenerative disease). Participants underwent a complete periodontal examination (plaque index and probing depth), and clinical subgingival sampling was conducted to quantify *Porphyromonas gingivalis* counts. The association between PKD and PE was evaluated through univariate and binary logistic regression analyses.

**Results:**

A high prevalence of PE (73.2%) was observed among individuals with PKD [OR=3.99 (2.00-7.59); p<0.001]. Variables that were significantly retained in the final binary logistic regression model for the occurrence of PKD were: PE (OR=3.23; p=0.003), mean *P. gingivalis* counts (OR= 3.39; p=0.036), lower number of teeth (OR= 2.21, p<0.001), low frequency of toothbrushing/day (OR=1.95; p=0.006), and bleeding on probing (OR=2.45; p<0.001). The significant positive correlations observed between *P. gingivalis* counts and clinical periodontal parameters were considered moderate to strong in individuals with PKD.

**Conclusions:**

A strong association between PKD and PE was demonstrated, with risk increasing in parallel with the severity of periodontal involvement. Furthermore, individuals with PKD exhibited significantly higher levels of P. gingivalis compared to controls.

## Graphical abstract

**Figure:**
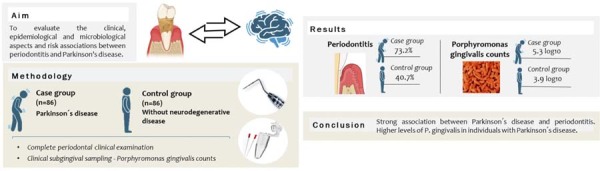

## Introduction

Periodontitis (PE) is one of the most common oral infectious diseases and is associated with the formation of a highly pathogenic biofilm.^1^ The development of this bacterial biofilm can initiate gingival inflammation. However, the onset and progression of PE depend on dysbiotic ecological changes in the oral microbiome, which are modulated by the host’s immune response and influenced by environmental factors.^2^ PE is an inflammatory condition identified as a source of numerous inflammatory mediators in the bloodstream that can exacerbate several systemic conditions. Over the past two decades, strong evidence has emerged supporting the idea that PE can negatively affect distant organs and tissues.^3^

Parkinson’s disease (PKD) is a progressive neurodegenerative disorder that affects the central nervous system, causing dysfunction in dopaminergic, noradrenergic, serotonergic, and cholinergic systems. Clinically, it manifests with altered motor and cognitive signs and symptoms; the most classic symptoms include tremors, rigidity, speech changes, memory loss, and sleep disorders.^4-8^

Sociodemographic characteristics (such as sex and age), as well as systemic factors like cardiovascular diseases, hormonal disorders, and harmful habits such as smoking, are considered common risk factors predisposing individuals to the onset and progression of both PE and PKD.^8^ Research in periodontology seeks to identify other potential risk factors contributing to the development of this oral disease and investigates how PE may influence the onset and progression of other diseases. Studies have suggested a possible link between PKD and PE, also related to chronic inflammation and dysbiosis.^5,8-12^

Most studies correlating oral health problems with PKD link the patient’s cognitive and motor impairment to difficulties in maintaining proper oral hygiene.^8,9,11,13^

Conversely, some studies suggest that pro-inflammatory proteins such as IL-1β, IL-6, and TNF-α, which are part of PE pathophysiology, may migrate through the bloodstream to neural tissues, causing or worsening neurodegeneration. Furthermore, lipopolysaccharides from periodontopathogenic bacteria such as *Porphyromonas gingivalis* have been detected in brain tissues, suggesting possible systemic dissemination originating from the oral cavity. These findings support the hypothesis that PE may be a contributing factor to neurodegenerative diseases such as PKD.^13-15^

Thus, we hypothesize that individuals with PKD have a higher prevalence and severity of PE compared to controls, as well as higher counts of *P. gingivalis*.

In this context, this study aimed to evaluate the clinical, epidemiological, and microbiological aspects (specifically *P. gingivalis* counts), as well as potential risk associations between PE and PKD through a case-control study.

## Methodology

This case-control study was conducted in Brazil at the Federal University of Minas Gerais (UFMG) from 2021 to 2025. Patient screening and recruitment took place at the Geriatrics and Gerontology Service and the Neurology outpatient clinic of the Jenny de Andrade Faria Institute for Older Adults and Women’s Health, located at the UFMG Clinical Hospital. Additionally, recruitment was carried out in nursing homes, private dental offices in Belo Horizonte, and within the UFMG institution itself. The study was approved by the UFMG Research Ethics Committee (CAAE: 48798321.0.0000.5149) and followed the 1975 Declaration of Helsinki as revised in 2013. All participants and/or their legal representatives were informed about the study procedures and signed a consent form before participating. The Strengthening the Reporting of Observational Studies in Epidemiology (STROBE) checklist was followed to standardize and control the report.

The case group consisted of individuals diagnosed with PKD, confirmed by experienced neurologists following diagnostic guidelines proposed by Póstuma, et al.^16^(2015). In addition, the Mini Mental State Examination (MMSE)^17^ was conducted on 65 patients to assess cognitive functions, including memory, orientation, attention, language, calculation, and visuospatial coordination, quickly and systematically. The total MMSE score ranges from 0 to 30, with the following interpretations: 27–30 points (normal cognitive function); 21–26 (mild cognitive impairment); 10–20 (moderate cognitive impairment); and <10 (severe cognitive impairment). The control group was composed of caregivers, family members, and staff from recruitment locations, all without any neurodegenerative disease, as determined by the same inclusion criteria applied to the cases.

Eligibility criteria for the total sample included individuals with or without PKD who were nonsmokers^18^ and non-diabetic^19^, male or female, aged 55 or older, without severe systemic conditions (ASA IV), with at least 10 teeth, and who had not undergone periodontal treatment in the last 6 months or used antimicrobials or anti-inflammatory drugs in the last 3 months.

Data were collected via anamnesis, including medical history to understand systemic health, dental history, pharmacological history, presence of harmful habits such as smoking and alcohol use, and systemic conditions such as cardiovascular disease and diabetes.

### Sample size

The sample size was estimated assuming an expected periodontitis prevalence of 60% in individuals with PKD compared to 30% in controls.^10^ The Fleiss method with continuity correction was used via OpenEpi software (OpenEpi, version 3.01, Boston, MA, USA). Based on a 0.05 significance level, 80% study power, and a 1:1 case-control ratio, a minimum of 84 cases and 84 controls were determined to be necessary.

### Examiner calibration

Inter- and intra-examiner Kappa concordance tests (APMM, AAC, FOC, NAG, VBO) were performed in a pilot study with 12 individuals for probing depth and clinical attachment level measurements, yielding values above 0.93. Intraclass correlation coefficients were greater than 0.90.

### Periodontal examination

Oral exams were conducted in dental chairs or beds using dental instruments, headlamps with adjustable focus, clinical mirrors, and UNC-15 millimeter probes (Hu-Friedy, Chicago, IL, USA). Each participant underwent a full-mouth periodontal exam, with parameters measured circumferentially at six sites per tooth. The highest value was recorded for the mesial, distal, buccal, and lingual/palatal sites. Parameters measured included probing depth (PD), clinical attachment level (CAL), bleeding on probing (BOP), and visible plaque index (VPI). Due to logistical management difficulties in the case groups, radiographs were not performed.

### Crevicular fluid sample collection

Microbiological analysis included 50% of the sample from each group; thus, 43 individuals per group were randomly selected (simple random sampling). In total, four samples were collected from periodontal sites with the greatest probing depth, following Cortelli, et al.^20^ (2015) recommendations.

Genomic DNA extraction from all samples was performed using the PureLink Genomic DNA Purification Kit (Invitrogen, Carlsbad, CA, USA ), according to the manufacturer’s instructions. Quantification of *P. gingivalis* was carried out using quantitative real-time polymerase chain reaction (qPCR) with specifically designed primers tested for specificity. Positive and negative controls were included in the analysis.

### Periodontitis diagnosis

This study used the 2017 classification proposed by the American Academy of Periodontology (AAP) and the European Federation of Periodontology (EFP) to characterize and stage periodontitis.^2^ Criteria were applied as follows: Stage I: ≥2 interproximal sites with CAL of 1 to 2 mm and PD ≤4 mm, with no tooth loss due to periodontitis; Stage II: ≥2 interproximal sites with CAL of 3 to 4 mm and PD ≤5 mm; and Stages III and IV (grouped): ≥2 interproximal sites with CAL ≥5 mm and PD ≥6 mm. Stage I periodontitis was not recorded, as it represents a borderline condition between health and disease.

The extent of periodontitis was classified as localized (≤30% of teeth affected) or generalized (>30% of teeth affected).^2^

**Figure 1:**
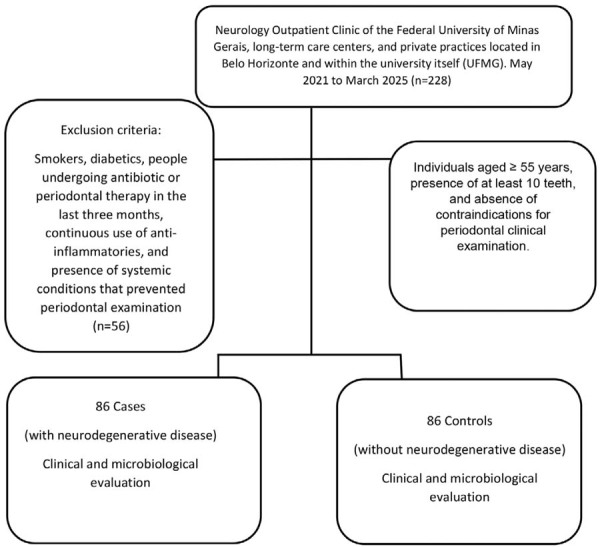
Sampling strategy flowchart.

A flowchart of the study is shown in Figure 1.

### Statistical analysis

Data normality was tested with the Kolmogorov-Smirnov test. Case and control groups were compared for age, sex, disease duration, family income, body mass index, education level, alcohol consumption, tooth brushing frequency, use of dental floss, number of teeth, and *P. gingivalis* counts (n=43 per group). Periodontal parameters (VPI, PD, CAL, and BOP) were summed across all sites per individual and expressed as means, standard deviations, and/or percentages. Comparisons used chi-square and Mann-Whitney tests as appropriate.

The final multivariate model for PKD occurrence was assessed using binary logistic regression. Independent variables with p<0.05 were retained in the final models. Variables included in the multivariate models were checked for independence by evaluating collinearity.

Correlation between *P. gingivalis* counts and periodontal parameters in case and control groups was assessed using Spearman’s correlation coefficient.

All statistical analyses were performed using R software R (Core Team - version 4.5.0; 2025; Vienna, Austria)

## Results

The sample comprised 172 individuals, including 122 men and 50 women. Patients with PKD had a mean disease duration of 9.4 years (±3.9) and a mean MMSE score of 14.2 (±2.9), indicating moderate to severe cognitive impairment. The use of antidepressants and anxiolytics among PKD patients was notable, at 59.3% (n=51) and 41.8% (n=36), respectively. Despite the lack of matching, the groups were considered homogeneous in important variables such as sex, age, alcohol consumption, use of dental floss, educational level, and family income, with significant differences found in lower BMI (p=0.008) and less frequent daily toothbrushing (p<0.014) in the case group compared to controls (Table 1).

**Table 1:** Characterization of study groups.

Variables	Controls	PKD
	(n=86)	(n=86)
Age (years) (p=0.117)*	61.9± 4.3	63.2 ± 5.1
**Disease Period (years)**	NA	9.4 ±3.9
**MMSE**^**‡**^	NA	14.2 ± 2.9
Body mass index (p=0.008)*	26.2 ± 2.4	25.3 ±1.8
**Sex**^**†**^		
Male	60 (69.7%)	62 (72.1%)
Female	26 (30.3%)	24 (27.9%)
	-	OR= 1.34 (0.68-2.65)
		p=0.201
**Alcohol consumption**^**†**^		
No	41 (48.0%)	63 (73.2%)
Yes	45 (52.0%)	23 (26.8%)
	-	OR=1.4 (0.91-2.03)
		p<0.042
**Dental floss use**^**†**^		
No	36 (41.8%)	57 (66.3%)
Yes	50 (58.2%)	29 (33.7%)
	-	OR=1.2 (0.72–2.10)
		p=0.194
**Frequency of toothbrushing / day**^**†**^		
1 to 2 times	47 (55.0%)	61 (70.9%)
≥3 times	39 (45.0%)	25 (29.1%)
	-	OR= 2.02 (1.07-3.80)
		p<0.014
**Family income**^**†**^		
<3 Brazilian Minimum Wage (BMW)	35 (40.7%)	49 (56.9%)
≥ 3 BMW	51 (59.3%)	37 (43.1%)
	-	OR=0.95 (0.52–1.74)
		p=0.439
**Educational level**^**†**^		
<8 years	59 (69.1%)	54 (62.6%)
≥8 years	27 (30.9%)	32 (37.4%)
	-	OR= 0.54 (0.41–1.45)
		p=0.214

* Mann-Whitney test; †Chi-squared test; ‡MMSE= Mini-Mental State Examination (Data from 65 participants); PKD = Parkinson’s disease; NA = not applicable; BMW = Brazilian minimum wage (equivalent to ~250 US$); OR= Odds ratio 95%CI

PKD = Parkinson’s disease; Mean±standard deviation. PI= Plaque Index; PD = probing depth; CAL = clinical attachment level; BOP = bleeding on probing; *Chi-square test; ^†^Mann-Whitney test. Significant p-values are shown in bold.

Table 2 shows the occurrence, severity, and extent of periodontitis between the study groups. A high prevalence of periodontitis (stages II + III + IV) was observed in the PKD group (73.2%), compared to the control group (40.7%). In the univariate analysis, the case group had approximately four times higher odds of periodontitis occurrence [OR=3.99 (2.00–7.59; p<0.001)] compared to controls. Moreover, individuals in the case group showed a significantly greater occurrence of moderate periodontitis [stage II; OR=2.41 (1.13–5.17); p=0.012] and severe-advanced periodontitis [stages III and IV; OR=6.65 (2.99–14.77); p<0.001] compared to controls. With regard to the extent of PE, the localized form was observed in 60.0% of cases and 44.4% of controls (p=0.002), while the generalized form was found in 55.6% of cases and 40.0% of controls (p<0.001).

**Table 2:** Occurrence, severity and extent of periodontitis among study groups.

Variables	Controls	PKD
	(n=86)	(n=86)
**Periodontitis**		
(stage II + III + IV)		
No	51 (59.3%)	23 (26.7%)
Yes	35 (40.7%)	63 (73.2%)
	-	OR= 3.99 (2.00–7.59) p<0.001*
**Periodontitis severity**		
Stage II (moderate)	22 (62.9%)	24 (38.0%)
	-	OR= 2.41 (1.13-5.17)
		p=0.012*
Stages III and IV (severe and advanced)	13 (37.1%)	39 (62.0%)
	-	OR=6.65 (2.99-14.77)
		p<0.001*
**Periodontitis extent**		
Localized	21 (60.0%)	28 (44.4%)
	-	OR= 2.95 (1.39-6.25)
		p=0.002*
Generalized	14 (40.0%)	35 (55.6%)
	-	OR= 5.54 (2.5-12.2)
		p<0.001*

*Chi-squared test. OR = odds ratio; 95%CI = confidence interval; Significant p-values are shown in bold.

Regarding periodontal status, individuals in the case group presented significantly worse clinical periodontal parameters than those in the control group: lower number of teeth, higher plaque index (PI), greater percentage of sites with BOP, greater PD of 4–6 mm and ≥6 mm, and greater CAL of 3–5 mm and >5 mm (Table 3).

**Table 3:** Periodontal status and mean P. gingivalis counts in the study groups.

Variables	Controls	PKD	p
	(n=86)	(n=86)	
**Number of teeth**^**†**^	22.8 ± 2.7	18.2±4.9	<0.001
**PI**^**†**^	35.7± 10.9	56.7±12.2	<0.001
**BOP**^**†**^	33.1±14.9	46.4±17.2	<0.001
Number of sites with PD*	7.843	6.26	
≤ 3mm	4.800 (61.2%)	3.142 (50.2%)	
4–6 mm	2.847 (36.3%)	2.767 (44.2%)	<0.001
> 6mm	196 (2.5%)	351 (5.6%)	
**Mean PD (mm)**^**†**^	1.8±0.75	3.5±0.67	<0.001
Number of sites with CAL*	7.71	6.194	
≤ 3 mm	3.230 (41.9%)	2.273(36.7%)	
4–5 mm	4.079 (52.9%)	3.444 (55.6%)	0.012
> 5mm	401 (5.2%)	477 (7.7%)	
**Mean CAL (mm)**^**†**^	2.7±0.7	3.8±1.3	<0.001
	N=43	N=43	
**Mean *P. gingivalis* counts (mean log10 ± s.d.)**^**†**^	3.9±1.9	5.3±2.5	0.012
**Periodontitis**^**†**^			
Yes			
No			
	2.5±1.3	3.9±1.7	<0.001
	1.4±0.6	2.4±0.8	<0.001

Parkinson's disease and *Porphyromonas gingivalis* levels: a case-control study

In terms of the presence of *P. gingivalis*, patients with PKD showed significantly higher mean counts, both in the subgroup with periodontitis and without [3.9±1.7 and 2.4±0.8 log10, respectively (total: 5.3±2.5)], compared to controls with and without periodontitis [2.5±1.3 log^10^ and 1.6±0.6 log^10^; respectively (total: 3.9±1.9)] (Table 3).

The final binary logistic regression models for PKD occurrence are shown in Table 4. Variables that were significantly retained in the final model for occurrence of PKD were: periodontitis (OR=3.23; p=0.003), bleeding on probing (OR=2.45; p<0.001), lower number of teeth (OR=2.21; p<0.001), mean *P. gingivalis* counts (OR=3.39; p=0.036), and low frequency of tooth brushing/day (OR=1.95 ; p=0.006). The model maintained good overall discriminatory ability (sensitivity and specificity).

**Table 4:** Final Multivariate binary logistic regression for the occurrence of Parkinson’s disease.

	Variables	Coefficient	OR (95% CI)	p
Final model				
Low frequency of toothbrushing / day)		0.723	1.95 (1.39 – 2.96)	0.006
Lower number of teeth		1.837	2.21 (2.01 – 2.15)	<0.001
Bleeding on probing		0.956	2.45 (1.62 – 2.45)	<0.001
Mean *P. gingivalis* counts		1.230	3.39 (1.13 – 1.92)	0.036
Periodontitis		0.587	3.23 (1.92 – 5.90)	0.003
Constant		-2.72	-	<0.001

OR = Odds ratio; 95%CI = Confidence interval. Final model: Pseudo R2 = 0.269; sensitivity = 74.81%; specificity = 85.34%; area under the ROC curve = 0.761. Significant p-values are shown in bold.

In the control group, moderate correlations were observed between *P. gingivalis* and PD > 4 mm (r=0.43; p=0.023). The correlation between *P. gingivalis* and BOP was weak (r=0.16), though significant (p=0.001). In the case group, all significant correlations observed between *P. gingivalis* counts and periodontal clinical parameters were considered moderate to strong (Table 5).

**Table 5:** Correlation between P. gingivalis counts and periodontal parameters in case and control groups.

Groups	^**†**^Periodontal Parameters		*P. gingivalis*	
			r*	p
**Case (PKD) (n=43)**	IP	0.75	0.80	**<0.001**
	PD>4 mm	0.72	0.82	**<0.001**
	CAL>3 mm	0.43	0.73	**<0.001**
	BOP	0.72	0.80	**<0.001**
**Control (n=43)**	IP	0.52	0.48	**0.012**
	PD>4 mm	0.43	0.64	**0.023**
	CAL>3 mm	0.22	0.52	0.062
	BOP	0.16	0.42	**0.001**

* Spearman correlation coefficient; ^†^ Mean number of sites.

In the case group, MMSE scores were distributed as follows: mild impairment (24.0%), moderate impairment (62.8%), and severe impairment (13.2%). Additionally, lower mean MMSE scores were associated with stage III and IV periodontitis, that is, greater severity of periodontitis (Mann Whitney U test, p=0.028).

## Discussion

Periodontitis ranks 11th among the most prevalent diseases globally.^21^ This global concern is expected to increase in the near future, leading to a need for better understanding of its outcomes related to systemic health.^22^ PKD is an advanced neurodegenerative disease characterized by dementia and cognitive decline and impairment, often resulting in morbidity and mortality among the older adults.^7,21^ These diseases are also likely to become more prevalent due to increased longevity and continued exposure to deleterious risk factors in today’s global population.^23^

This study showed a higher prevalence of periodontitis among individuals with PKD (73.2%) compared to controls (40.7%). Furthermore, advanced stages of periodontitis (III and IV) were significantly more frequent in the PKD group (62.0%) than in the control group (37.1%). These findings are very similar to those reported by Lyra, et al.^11^ (2022), who found a periodontitis prevalence of 75% in PKD patients, with 46.4% in the most advanced stages.

Overall, supporting these findings, most observational studies have demonstrated a strong association between neurodegenerative diseases and periodontitis, showing a much higher prevalence of periodontitis in individuals with neurodegenerative diseases compared to those without.^5,8-11,13,24,25^ However, some studies have failed to demonstrate a link between the two conditions^9,26-28^ or have shown only a modest association.^29^

Individuals with PKD had a significantly higher PI than controls (56.7±12.2 *vs*. 35.7±10.9; p<0.001), similar to findings from other studies.^5,8,11,29^ We hypothesize that, given the MMSE scores indicated moderate to severe cognitive impairment (mean score of 14.2±2.9), this condition may have impacted the effectiveness of oral hygiene practices. Additionally, our results showed a strong positive correlation between PI and *P. gingivalis* counts, which may also be linked to reduced motor function in individuals with PKD.

Moreover, the literature highlights challenges in maintaining oral hygiene in PKD patients. Besides limitations due to cognitive and motor decline, socioeconomic and emotional factors may also hinder access to proper dental care.^5,8,11,30-32^ However, a recent study reported no significant association between poor oral health and the risk of PKD in men or women.^8^

In this study, PKD patients reported lower frequency of daily toothbrushing, a factor that may contribute to poor oral health. Multivariate logistic regression identified low frequency of daily brushing (OR=1.95; p=0.0006) as a factor associated with PKD. It is important to note that individuals with neurodegenerative diseases showed significantly higher PI, and that BOP was strongly associated with PKD.

In recent years, interest in the relationship between PKD and periodontitis has increased significantly. The biological plausibility of this association may be based on: (1) shared features such as a hyper-inflammatory phenotype and pathogenic similarities in risk factors, immunogenetics, and tissue destruction pathways; (2) systemic infections generating a pro-inflammatory state that may lead to blood-brain barrier disruption and allow inflammatory cells to penetrate the CNS and activate microglia;^25^ (3) neuroinflammation compatible with infection, including inflammasome activation, complement system activation, and cytokine profile changes in neurodegenerative patients, which may be potentially exacerbated by polymicrobial infections;^33^ and (4) periodontitis aggravating systemic diseases such as diabetes and cardiovascular conditions through the release of inflammatory cytokines and reactive oxygen species from inflamed periodontal tissue, affecting other organs and systems. Chronic systemic inflammation may also lead to insulin resistance, which can accelerate atherosclerosis and contribute to dementia.^33^

Microbiological analysis revealed significantly higher levels of *P. gingivalis* in the PKD group (5.3±2.5 log^10^) compared to controls (3.9±1.9 log^10^; p=0.012). Moderate to strong positive correlations were observed between *P. gingivalis* levels and clinical periodontal parameters such as PI, PD, and BOP, especially in the PKD group. Ermini, et al.^34^ (2024) conducted post-mortem neuronal tissue analysis of PKD individuals using immunohistochemistry and fluorescence microscopy and detected gingipains, proteolytic enzymes secreted by *P. gingivalis*, located in α-synuclein aggregates in dopaminergic neurons of the substantia nigra, a key region in PKD pathology. This provides direct experimental evidence of *P. gingivalis* involvement in PKD pathophysiology.

Another relevant result was that patients with PKD showed significantly higher mean *P. gingivalis* counts, both in the subgroup with PE and without [respectively, 3.9 ± 1.7 and 2.4±0.8 log10; (total: 5.3±2.5)], compared to controls with and without PE [respectively, 2.5±1.3 log^10^ and 1.6±0.6 log^10^; (total: 3.9±1.9)], suggesting a dysbiosis of *P. gingivalis* in individuals with PKD independent of periodontal condition.

Li, et al.^35^ (2022) conducted a cross-sectional observational study comparing PKD individuals with mild cognitive impairment, PKD individuals without cognitive impairment, and healthy controls matched for periodontal status. They analyzed gingival crevicular fluid for *P. gingivalis* counts and the KGP II genotype (a lysine-specific protease linked to inflammation and neurodegeneration). *P. gingivalis* counts and KGP II presence were higher in individuals with PKD and mild cognitive impairment. The authors suggested a direct association between this bacterial load and cognitive decline, which may affect oral hygiene.

It is also worth noting the strong presence of risk variables such as frequent use of antidepressants and anxiolytics, which may cause xerostomia and affect oral hygiene habits.^31^ A recent study showed that PKD patients report a higher prevalence of xerostomia or sialorrhea than controls.^36^

The final binary logistic regression model for the occurrence of PKD identified, in addition to lower frequency of daily toothbrushing, the following variables as significant predictors: periodontitis, mean *P. gingivalis* counts, lower number of teeth, and bleeding on probing. The model demonstrated good overall discriminatory capacity, balancing sensitivity and specificity.

Few studies report no differences in oral health conditions between patients with PKD and individuals without neurodegenerative diseases.^9,29,37^ On the other hand, several studies have reported a lower number of teeth in individuals with PKD, which is attributed to reduced cognitive and motor abilities, poorer oral hygiene habits, and a higher prevalence of dental caries and periodontal disease.^3,5,15,25,27,38^ Supporting these findings, our results indicated that lower mean MMSE scores were associated with greater periodontitis severity.

However, Thomson and Barak^39^ (2021) proposed an interesting explanation, in which they applied a life course approach to offer a plausible and empirically supported rationale for the association between tooth loss and poorer cognitive function in older adults. Evidence from long-term cohort studies suggests that this association originates in childhood, particularly in early cognitive development. Individuals with higher cognitive functioning in childhood are more likely to maintain better oral health and to access routine dental care throughout life, leading to fewer lost teeth over time. They are also significantly more likely to retain better cognitive function in older age. In contrast, those with lower cognitive abilities in childhood tend to experience a higher disease burden and limited access to care, resulting in a progressive increase in tooth loss. Comparisons between these groups from as early as their mid-20s already reveal more missing teeth among those with lower childhood cognitive performance differences that become increasingly pronounced in later life.^39^

Some limitations of this study include the convenience sample, which may affect external validity, and the case-control design, which does not allow for temporal inference between PKD and PE. Observational studies cannot definitively establish the direction of the association. However, we minimized these effects by ensuring that the total sample had overall homogeneous risk variables. Other minor limitations include: (1) missing MMSE data for 24.4% of the sample and (2) microbiological analysis performed in 50.0% of the sample due to processing costs.

Nonetheless, some advantages should also be noted, such as:

a) the number of participants was consistent with the sample size calculation, increasing the statistical power of the study. It is important to emphasize the difficulty encountered by patients, family members, and caregivers in allowing periodontal examinations to be performed; b) the diagnosis of PKD was made by specialist physicians; c) the use of full-mouth periodontal examination, current diagnostic criteria for PE, and the exclusion of stage I periodontitis to avoid overestimating disease prevalence—recognizing that the quality of periodontal data and case definitions significantly influence the outcomes of association studies^40^; d) exclusion of individuals with diabetes and smokers, eliminating the strong confounding effects of these variables; and finally e) the microbiological assessment of *P. gingivalis* counts.

The microbiological results highlight the need for future longitudinal studies to investigate changes in the oral microbiota during the early stages of PKD and to monitor its progression over time. Preventive dental care targeting microbiota modulation may serve as a complementary strategy for individuals at risk of developing neurodegenerative diseases.

Our findings suggest that family members, physicians, caregivers, and physical therapists should encourage dental visits, preventive oral health measures, and improved hygiene practices for these vulnerable individuals. A more multidisciplinary approach to managing both periodontitis and PKD is needed to improve patient quality of life.^6^ These findings may serve as an important starting point for new confirmatory studies, preferably prospective and interventional in design with high methodological rigor, so that the causal relationship of this association can be well established.

Finally, this study supports the inclusion of periodontitis as a comorbidity associated with PKD, raising important questions about lifestyle, microbiological and genetic susceptibilities, and phenotypes in patients with both conditions, warranting further investigation. PE may become more common in older adults with neurodegenerative diseases due to increasing difficulties in maintaining oral hygiene.

## Conclusion

A strong association between PKD and PE was demonstrated, with the risk increasing in parallel with the severity of periodontal involvement. Furthermore, individuals with PKD exhibited significantly higher levels of *P. gingivalis* compared to controls. These findings reinforce the potential role of periodontal inflammation and microbial dysbiosis in the pathophysiology of PKD.
